# The ratio between the whole-body and primary tumor burden, measured on ^18^F-FDG PET/CT studies, as a prognostic indicator in advanced non-small cell lung cancer

**DOI:** 10.1590/0100-3984.2020.0054

**Published:** 2021

**Authors:** Felipe Renê Alves Oliveira, Allan de Oliveira Santos, Mariana da Cunha Lopes de Lima, Ivan Felizardo Contrera Toro, Thiago Ferreira de Souza, Bárbara Juarez Amorim, Aristoteles Souza Barbeiro, Elba Etchebehere

**Affiliations:** 1 Faculdade de Ciências Médicas da Universidade Estadual de Campinas (FCM-Unicamp), Campinas, SP, Brazil.

**Keywords:** Fluorodeoxyglucose F18, Positron-emission tomography/methods, Tomography, X-ray computed/methods, Carcinoma, non-small-cell lung/diagnosis, Tumor burden, Carcinoma, non-small-cell lung/mortality, Fluordesoxiglicose F18, Tomografia por emissão de pósitrons/métodos, Tomografia computadorizada/métodos, Carcinoma pulmonar de células não pequenas/diagnóstico, Sobrevivência, Carga tumoral, Carcinoma pulmonar de células não pequenas/mortalidade

## Abstract

**Objective:**

To determine whether the whole-body tumor burden, as quantified by ^18^F-fluorodeoxyglucose positron-emission tomography/computed tomography (^18^F-FDG PET/CT), is a prognostic indicator in advanced (stage III or IV) non-small cell lung cancer (NSCLC).

**Materials and Methods:**

This was a prospective study in which we evaluated ^18^F-FDG PET/CT staging parameters to quantify tumor burdens in patients with stage III or IV NSCLC. The following parameters were evaluated for the whole body (including the primary tumor) and for the primary tumor alone, respectively: maximum standardized uptake volume (wbSUV_max_ and tuSUV_max_); metabolic tumor volume (wbMTV and tuMTV); and total lesion glycolysis (wbTLG and tuTLG). To determine whether the ^18^F-FDG PET/CT parameters were associated with overall survival (OS) and progression-free survival (PFS), we evaluated the wbSUV_max_/tuSUV_max_, wbMTV/tuMTV, and wbTLG/tuTLG ratios.

**Results:**

^18^F-FDG PET/CT was performed for staging in 52 patients who were followed for a median of 11.0 months (mean, 11.7 months). The estimated median PFS and OS were 9.6 months and 11.6 months, respectively. In the univariate analysis, OS was found to correlate significantly with wbTLG (hazard ratio [HR] = 1.001; 95% confidence interval [95 CI]: 1.000-1.001; *p* = 0.0361) and with the wbTLG/tuTLG ratio (HR = 1.705; 95% CI: 1.232-2.362; *p* = 0.0013). In the multivariate analysis, only the wbTLG/tuTLG ratio was independently associated with OS (HR = 1.660; 95% CI: 1.193-2.310; *p* = 0.0027).

**Conclusion:**

The wbTLG/tuTLG ratio is an independent prognostic indicator of OS in advanced-stage NSCLC.

## Abstract (pt)

**Objetivo:**

Avaliar se a carga metabólica tumoral do corpo inteiro medida na tomografia por emissão de pósitrons/tomografia computadorizada com ^18^F-fluordesoxiglicose (^18^F-FDG PET/CT) é um indicador prognóstico em pacientes com câncer de pulmão de células não pequenas (CPCNP) em estágio avançado (estágio III ou IV).

**Materiais e Métodos:**

Avaliamos, prospectivamente, a carga tumoral na ^18^F-FDG-PET/CT de estadiamento em pacientes com CPCNP avançado. Os parâmetros avaliados do tumor primário (tu) e do corpo inteiro (wb) (incluindo o primário) foram: SUV máximo (wbSUV_max_ e tuSUV_max_), volume metabólico tumoral (wbMTV e tuMTV), glicólise total da(s) lesão(ões) (wbTLG e tuTLG), além das seguintes razões: wbSUV_max_/tuSUV_max_, wbMTV/tuMTV e wbTLG/tuTLG. Os parâmetros medidos na ^18^F-FDG-PET/CT, variáveis clínicas e patológicas foram correlacionados com a sobrevida global (SG) e a sobrevida livre de progressão (SLP).

**Resultados:**

^18^F-FDG-PET/CT foi realizada em 52 pacientes (tempos mediano/médio de sobrevida = 11,0/11,7 meses). A SLP mediana foi de 9,6 meses e a SG foi de 11,6 meses. Houve correlação significativa da wbTLG (*hazard ratio* [HR] = 1,001; intervalo de confiança de 95% [IC 95%]: 1,000-1,001; *p* = 0,0361) e wbTLG/tuTLG (HR = 1,705; IC 95%: 1,232-2.362; *p* = 0,0013) com a SG. Na análise multivariada, a razão wbTLG/tuTLG associou-se independentemente com a SG (HR = 1,660; IC 95%: 1,193-2,310; *p* = 0,0027).

**Conclusão:**

A razão wbTLG/tuTLG é um indicador prognóstico independente de SG em CPCNP avançado.

## INTRODUCTION

Lung cancer is the leading cause of death worldwide^(1,2)^. Non-small-cell lung cancer (NSCLC) accounts for 80% of all cases of lung cancer, and more than half of all patients with NSCLC have metastatic disease at the time of diagnosis, the 5-year survival rate for all stages combined being 18%^(2)^. For advanced NSCLC, the primary treatment modalities are chemotherapy and chemoradiotherapy^(1,2)^. Overall survival (OS) is low in NSCLC even in the earlier tumor-node-metastasis (TNM) stages, decreasing progressively as the TNM stage increases-from 50% in stage IA to 2% in stage IV^(2)^.

A TNM-based stage grouping of I-IV is a prognostic indicator in patients with lung cancer. However, TNM staging is not able to differentiate between stage III/IV patients with oligometastatic disease and patients with extensive metastatic NSCLC. In the routine clinical setting, the prognosis of stage IV NSCLC that presents as oligometastatic disease may not differ significantly from that of stage III NSCLC^(3)^.

In parallel with the TNM staging, metabolic parameters on ^18^F-fluorodeoxyglucose positron-emission tomography/computed tomography (^18^F-FDG PET/CT)-maximum standardized uptake value (SUV_max_), metabolic tumor volume (MTV), and total lesion glycolysis (TLG)-have been shown to be predictors of the risk of disease recurrence and death in patients with NSCLC and may be used in order to stratify such patients; those who are at a higher risk of recurrence or death may be candidates for treatments that are more aggressive^(4)^. However, the SUV_max_ provides information only on a single pixel within the tumor, rather than measuring the volume or heterogeneity of the metabolically active disease^(5)^. In patients with NSCLC in various stages, the analysis of SUV_max_ in conjunction with the volumetric parameters (MTV and TLG) has been shown to have greater prognostic value than does that of SUV_max_ alone^(4)^. The addition of the volumetric parameters improves the determination of the biologically relevant lesion volume, especially in NSCLC^(6)^. Although the volumetric parameters obtained from ^18^F-FDG PET/CT studies are especially significant prognostic factors for outcomes in patients with TNM stage I or II NSCLC^(7)^^-^^(9)^, the application of such parameters in advanced disease has not been extensively studied^(10)^.

As previously mentioned, TNM staging is not able to differentiate between patients with advanced (stage III or IV) oligometastatic NSCLC and those with extensive metastatic NSCLC. Therefore, our study aimed to determine whether the ^18^F-FDG PET/CT-determined whole-body tumor burden is a better prognostic indicator than is the TNM stage in patients with advanced NSCLC, specifically whether it can discriminate between the prognosis of patients with oligometastatic stage IV disease and that of those with extensive locoregional stage III disease.

## MATERIALS AND METHODS

### Patients

This was a prospective study in which we evaluated baseline ^18^F-FDG-PET/CT scans performed for staging between March 2016 and September 2017. The local institutional review board approved the study (Reference no. 53952516.1.0000.5404). All participating patients and professionals gave written informed consent.

All patients with histologically confirmed stage III or IV NSCLC were included in the analysis, as were those in whom the histopathology was not able to specify the type of NSCLC. All of the patients underwent standard treatment with platinum-based chemotherapy, with or without radiotherapy (included only for patients with stage III disease), with subsequent clinical follow-up. Patients under 18 years of age were excluded, as were those who had had another neoplasm (except nonmelanoma skin cancer) in the last five years and those with uncontrolled diabetes mellitus, as well as women who were pregnant or lactating.

### ^18^F-FDG-PET/CT acquisition

All patients fasted for 6 h before undergoing ^18^F-FDG-PET/CT, which involved the injection of 4.44 MBq/kg of ^18^F-FDG. In all cases, the fasting serum glucose level was below 180 mg/dL. The studies were performed in a 64-slice PET/CT scanner (Biograph TruePoint 64; Siemens Medical Solutions, Knoxville, TN, USA) 60 min after radiotracer injection. The CT parameters included 5 mm axial reconstruction, with a tube voltage of 120 kV or automatic tube voltage selection (CARE Dose 4D; Siemens Healthcare, Forchheim, Germany). The PET images were acquired from the head to upper thighs in three-dimensional mode with 90 s/bed position, without contrast administration. 

### Image analysis and parameters used for quantification

**Figure 1.**
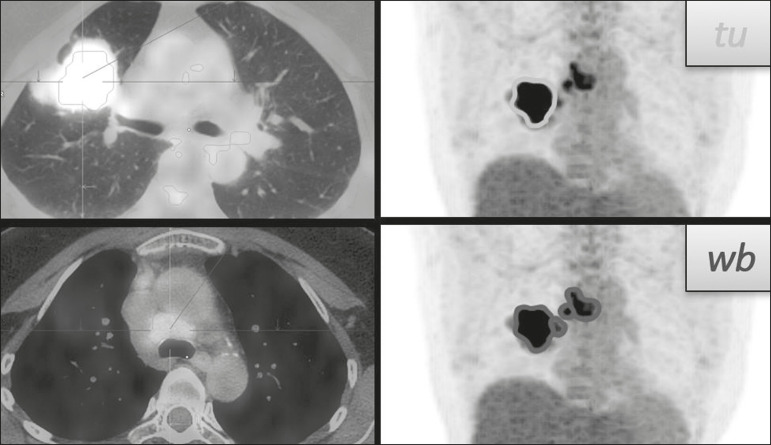
^18^F-FDG PET/CT in a patient with advanced NSCLC, showing the VOIs for the tuTLG (upper) and wbTLG (lower).

Two experienced observers, working independently, performed the quantitative visual analyses of all images. The final quantification was defined by consensus. On ^18^F-FDG PET/CT, the semiquantitative parameters evaluated were whole-body SUV_max_ (wbSUV_max_, obtained from all metastatic sites including the primary lesion) and SUV_max_ of the primary tumor (tuSUV_max_), whereas the quantitative parameters were whole-body MTV (wbMTV, including all metastatic sites and the primary lesion), primary tumor MTV (tuMTV), whole-body TLG (wbTLG, including all metastatic sites and the primary tumor), and primary tumor TLG (tuTLG), as depicted in Figure 1. We also calculated the wbSUV_max_/tuSUV_max_, wbMTV/tuMTV, and wbTLG/tuTLG ratios. 

All semi-automated assessments were performed with a semi-automated multifocal segmentation tool (Syngo.via MM Oncology; Siemens Medical Solutions, Knoxville, TN, USA). We established a three-dimensional isocontour threshold of 50% of the single-pixel SUV_max_ to delineate the tumor contours with a lower-limit cutoff SUV_max_ of 2.5 to allow the software to calculate tumor burden of the primary and metastatic lesions^(11)^^-^^(13)^. After the threshold selections were established, the software automatically generated volumes of interest (VOIs) for any structure with an SUV greater than the pre-established SUV_max_ threshold selection. It was necessary, at this point, to verify that every VOI delineated corresponded to a tumor site; if not, the VOI in question was manually excluded. Subsequently, the tumor burden was determined according to the selected tumor-related areas and the software automatically provided the ^18^F-FDG PET/CT parameters for the whole-body tumor burden.

### Statistical analyses

Statistical analyses were performed with the SAS System, version 9.4 for Windows (SAS Institute Inc., Cary, NC, USA). Cox regression analysis examined the effects that the whole-body tumor burden parameters and other clinical and pathological variables-age, sex, Eastern Clinical Oncology Group (ECOG) performance status, histology, and TNM stage-had on OS and progression-free survival (PFS). The correlation between wbMTV and wbTLG was analyzed with Spearman’s correlation coefficient.

Initially, the individual effects of variables on the outcomes were examined by univariate analyses. Subsequently, a forward and backward stepwise procedure was performed, and variables with *p*-values < 0.05 were selected for the multivariate analysis. Values of *p* ≤ 0.05 were considered statistically significant.

## RESULTS

We evaluated 52 baseline ^18^F-FDG PET/CT scans performed for staging in 52 patients (mean age, 64.2 years; 24 women and 28 men). The median and mean follow-up times were 11.0 and 11.7 months, respectively, and the estimated median PFS and OS were 9.6 and 11.6 months, respectively. None of the patients were lost to follow-up.

During follow-up, there was disease progression in 31 (59.7%) of the patients and 26 (50.0%) died. Table 1 shows the demographic and clinical characteristics of all patients; Table 2 describes the PFS and OS data; and Table 3 shows the distribution of the SUV, MTV, and TLG parameters.

**Table 1:** Demographic and clinical characteristics of the patients evaluated.

Patient characteristic	(N = 52)
Sex, n (%)	
Male	28 (53.8)
Female	24 (46.8)
Smoker, n (%)	
Yes	45 (86.54)
No	7 (13.46)
Histological type, n (%)	
Adenocarcinoma	25 (48.1)
Squamous cell carcinoma	19 (36.5)
NSCLC (undetermined)	8 (15.3)
Tumor stage, n (%)	
III	23 (44.2)
IV	29 (55.8)
ECOG score, n (%)	
0	3 (5.7)
1	42 (80.7)
2	6 (11.5)
3	1 (1.9)
PFS, n (%)	
Yes	29 (58.0)
No	21 (42.0)
OS, n (%)	
Yes	26 (50.0)
No	26 (50.0)

**Table 2:** Survival or follow-up time for PFS and OS among the patients evaluated (N = 52).

Survival	Mean ± SD (range)
PFS (months)	9.59 ± 6.94 (0.57-25.67)
OS (months)	11.61 ± 7.36 (1.03-26.33)

**Table 3:** ^18^F-FDG-PET/CT parameters among the patients evaluated (N = 52).

Parameter	Mean ± SD (range)
tuSUV_max_	22.3 ± 9.77 (8.3-53.0)
WbSUV_max_	23.0 ± 9.76 (8.3-53.0)
tuMTV (mL)	46.0 ± 34.63 (2.2-197.5)
wbMTV (mL)	76.7 ± 62.61 (12.4-305.7)
tuTLG (mL)	547.9 ± 428.40 (27.8-1,769.0)
wbTLG (mL)	777.9 ± 734.08 (60.2-3,566.3)
wbSUV_max_/tuSUV_max_ ratio	1.04 ± 0.14 (1.0-1.80)
wbMTV/tuMTV ratio	1.86 ± 1.24 (1.0-7.13)
wbTLG/tuTLG ratio	1.58 ± 1.24 (1.0-8.65)

To evaluate the effects that the tuSUV_max_, wbSUV_max_, wbSUV_max_/tuSUV_max_ ratio, tuMTV, wbMTV, wbMTV/tuMTV ratio, tuTLG, wbTLG, wbTLG/tuTLG ratio, performance status, and clinical stage have on OS, we applied a univariate Cox proportional-hazards model, including all of the same variables as the full model, and subsequently used a forward stepwise selection to construct the final model. The multivariate analysis included only the variables that achieved significance in the univariate analysis.

In the univariate analysis, OS was found to correlate significantly with the wbMTV (hazard ratio [HR] = 1.008; 95% CI: 1.002-1.014; *p* = 0.0076), wbTLG (HR = 1.001; 95% CI: 1.000-1.001; *p* = 0.0361), and wbTLG/tuTLG ratio (HR = 1.705; 95% CI: 1.232-2.362; *p* = 0.0013). Because all MTV and TLG parameters are sub-products of each other and therefore strongly associated (*p* < 0.0001), only TLG-based parameters were included in the multivariate analyses. In the multivariate analysis, only the wbTLG/tuTLG ratio was independently associated with OS (HR = 1.660; 95% CI: 1.193-2.310; *p* = 0.0027). None of the other tumor burden parameters (wbSUV_max_, tuSUV_max_, tuMTV, tuTLG, wbSUV_max_/tuSUV_max_ ratio, or wbMTV/tuMTV ratio), sex, age, performance status, histological subtype, TNM stage, or treatment type was found to be associated with PFS or OS in patients with advanced NSCLC (Table 4).

**Table 4:** Analyses of the predictors of OS.

Analysis	HR (95% CI)	*P*-value
Univariate		
ECOG performance status score		
0-1 vs. 2-3	0.806 (0.252-2.919)	0.806
Histology		
Adenocarcinoma vs. squamous cell carcinoma	1.065 (0.471-2.407)	0.800
TNM stage		
T (3 vs. 4)	1.174 (0.489 - 2.819)	0.719
N (2 vs. 3)	1.263 (0.567-2.812)	0.567
M (0 vs. 1)	1.866 (0.853-4.082)	0.118
Clinical stage		
III vs. IV	2.064 (0.894-4.767)	0.089
Volumetric parameters		
tuSUV_max_	1.002 (0.970-1.036)	0.890
wbSUV_max_	1.007 (0.975-1.040)	0.663
tuMTV	1.002 (0.992-1.012)	0.654
wbMTV	1.008 (1.002-1.014)	0.007
tuTLG	1.000 (0.999-1.001)	0.964
wbTLG	1.001 (1.000-1.001)	0.036
wbSUV/tuSUV ratio	6.725 (0.814-55.533)	0.076
wbMTV/tuMTV ratio	1.179 (0.881-1.577)	0.268
wbTLG/tuTLG ratio	1.705 (1.232-2.362)	0.013
Multivariate		
Volumetric parameters		
wbTLG/tuTLG ratio	1.660 (1.193-2.310)	0.002

## DISCUSSION

In this prospective study, we have demonstrated that, in patients with advanced (stage III or IV) NSCLC, the whole-body metabolic tumor burden determined on a baseline ^18^F-FDG PET/CT performed for staging seems to be a strong, independent imaging biomarker to predict OS, better than the clinical evaluation of the primary tumor itself. The wbTLG/tuTLG ratio was found to be the best predictor of OS in our patient sample.

**Figure 2.**
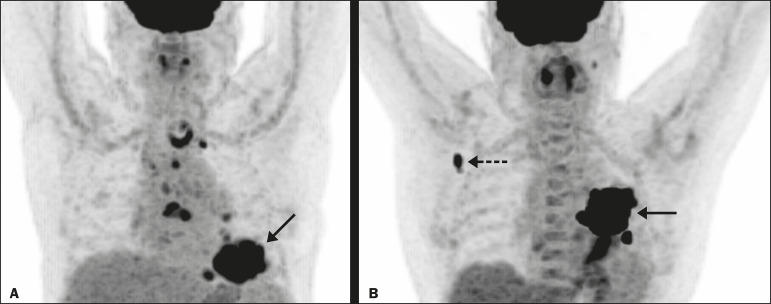
Examples of the importance of the tumor burden, calculated from ^18^F-FDG PET/CT imaging, as a prognostic indicator. **A:** A patient with stage IIIB NSCLC. The primary tumor (arrow) shows intense uptake (SUV = 13.2), and the multiple mediastinal lymph node metastases have intense hypermetabolism. **B:** A patient with stage IV NSCLC. The primary tumor (arrow) shows intense uptake (SUV = 47.2), and the patient was classified as having stage IV disease due to a contralateral axillary lymph node metastasis (dotted arrow). According to the TNM staging, the estimated 5-year OS rate was 19% for the patient depicted in **A** and 6% for the patient depicted in **B**. The wbTLG/tuTLG ratio was 1.27 for the patient in **A** and 1.01 for the patient in **B**. The patient in **A** had a higher tumor burden and died 11.57 months after diagnosis, whereas the patient in **B**, despite having been classified as having stage IV disease, was alive 25.7 months after diagnosis. Therefore, the wbTLG/tuTLG ratio, which is an independent prognostic indicator, may be a more accurate tool for assessing OS, which is especially useful in oligometastatic NSCLC.

In advanced NSCLC, the metabolism of the primary lesion, the TNM stage, and clinical parameters, although useful in the overall risk stratification, does not reflect the differences in survival when discriminating between patients with extensive metastatic disease and those with oligometastatic disease. The wbTLG/tuTLG ratio has the advantage of reflecting the relationship between and comparing the overall burden of disease and that of the primary tumor. Therefore, it seems reasonable to believe that this ratio reflects the aggressiveness of the disease because it reflects the capability of the tumor to spread in comparison with the burden of the primary lesion. In advanced NSCLC, a part of the tumor load, or even the primary tumor burden, is due to metastases, and the primary lesion alone (tuTLG) therefore does not adequately demonstrate the actual tumor burden, as demonstrated in Figure 2.

In our study, the univariate analyses showed that wbTLG and wbMTV were highly significant predictors of OS (*p* < 0.05 for both). Our findings corroborate those of other studies in which patients with a high MTV or TLG were found to be at a higher risk of adverse events and death^(14)^. Therefore, the volumetric parameters are more reliable parameters^(15)^ and should be used, as complements to the SUV, as incremental predictors of survival and events in patients with advanced NSCLC^(8,14)^. However, in the present study, they were not strong independent predictors, most likely because of the small number of patients evaluated, the narrow confidence intervals, and the low HRs.

As in other studies of patients with NSCLC^(9,16)^, the majority of our patients were male and adenocarcinoma was the predominant histological type^(15,17)^. However, unlike what has been reported in most such studies, in which the performance status of the patients is typically good (ECOG score = 0), nearly 92% of our patients had an ECOG score of 1 or 2, because they all had advanced NSCLC, which could account for the fact that there was no significant difference between those with stage III disease and those with stage IV disease. As expected on the basis of the literature^(9)^, age, performance status, histological subtype, TNM stage, and treatment type were not predictive of PFS or OS in our sample of patients with advanced NSCLC. Likewise, another study of patients with NSCLC showed that TLG was associated with both PFS and OS more strongly than were other variables, such as smoking status, performance status, and histological type, which have previously been reported to correlate significantly with patient survival^(18)^.

In most of our patients, the SUV_max_ was highest in the primary lung lesion, being highest in metastatic lesions in only a few cases, the tuSUV_max_ and wbSUV_max_ therefore being coincident (given that the wbTLG includes the primary lesion). Analysis of the SUV in isolation has limitations. Isolated SUVs may vary according to blood glucose level, fasting time, and uptake time, as well as the methods employed for attenuation correction and reconstruction.

The clinical importance of SUV_max_ as a prognostic factor is still unclear^(8)^. In advanced NSCLC, FDG uptake in the primary tumor alone does not correlate significantly with survival^(5,9,14)^. However, a meta-analysis including a collective total of 5,807 surgical patients with NSCLC demonstrated that high SUV_max_, MTV, and TLG predicted a higher risk of recurrence or death^(19)^. In contrast, another meta-analysis showed that the pre-radiotherapy and post-radiotherapy primary tumor SUV_max_ could predict the outcome of NSCLC patients treated with radiotherapy, because patients with a higher pre-radiotherapy SUV_max_ seemed to have a shorter OS and less local control^(20)^. Although some of our patients underwent radiotherapy, we did not find a correlation between the pre-radiotherapy SUV_max_ and survival.

The present study involved the use of semi-automated observer-independent software and, unlike most studies on this topic, had a prospective design. However, it has some limitations. First, the lesions were defined by using a threshold method. The choice of the threshold may influence the measurement of tumor volume, the mean SUV, and the wbTLG. We used 50% of the SUV_max_, which is a commonly adopted threshold^(9,21,22)^, to determine the tumor volume and then evaluated the results with the fused CT images to decide if further adjustment of the threshold was needed. Therefore, although the method is semi-automated, regions of physiological uptake were manually excluded. In addition, because this was a prospective study, it had a relatively small number of patients and a relatively short follow-up period. Furthermore, although all of our patients underwent chemotherapy, only those with stage III disease also underwent radiotherapy. Therefore, PFS and OS may also have been influenced by the treatment strategy employed. Moreover, the wbTLG/tuTLG ratio is new in the literature and has some intrinsic limitations, mainly the inability to differentiate between a high primary tumor burden with a high metastatic tumor burden and a low primary tumor burden with a low metastatic tumor burden.

Further studies are needed in order to determine standard cutoff values and delineation methods for predicting prognosis using this parameter. The wbTLG/tuTLG ratio should be tested in other advanced malignant FDG-avid neoplasms to prove its utility.

## CONCLUSION

In patients with advanced (stage III or IV) NSCLC, the wbTLG/tuTLG ratio measured on baseline ^18^F-FDG PET/CT performed for staging seems to be a strong, independent imaging biomarker, better than evaluation of the primary tumor in isolation, to predict OS.
